# Metachronous serous endometrial intraepithelial carcinoma and serous peritoneal carcinoma: analysis of probable independent lesions

**DOI:** 10.1186/s13000-016-0585-0

**Published:** 2016-11-14

**Authors:** Mitsuko Furuya, Teiko Sato, Reiko Tanaka, Masafumi Yamamoto, Naho Ruiz Yokota, Etsuko Miyagi

**Affiliations:** 1 Department of Molecular Pathology, Yokohama City University Graduate School of Medicine, 3-9 Fukuura, Kanazawa-ku, Yokohama, 236-0004 Japan; 2 Departments of Pathology, Keiyu, Hospital, Yokohama, Japan; 3 Medical Mycology Research Center, Chiba University, Chiba, Japan; 4 Department of Gynecology, Yokohama City University Graduate School of Medicine, Yokohama, Japan

**Keywords:** Superficial uterine serous carcinoma (SEIC), Peritoneal serous carcinoma, p53, Immunohistochemistry, Somatic mutation, Case report

## Abstract

**Background:**

Uterine serous endometrial intraepithelial carcinoma (SEIC) is an immediate precursor of invasive carcinoma. The majority of stage IA SEICs are curable, but those with latent peritoneal metastasis and/or capillary lymphatics invasion may have poor prognoses Careful pathologic staging is thus needed to predict the risk of recurrence and to determine postoperative therapeutic strategies.

**Case Presentation:**

A 71-year-old woman was hospitalized for the treatment of peritoneal carcinoma. She had undergone total hysterectomy and bilateral salpingo-oophorectomy due to SEIC (stage IA) at age 63 years, and had received medical check-ups every year since. Elevated serum CA125 (184 U/mL) was detected for the first time 8 years after surgery. A thorough workup revealed no potential primary lesion other than that in the peritoneum. Tumor reduction surgery was performed. Histologic analysis of the peritoneal lesion was high-grade serous carcinoma. The peritoneal carcinoma was diffusely immunostained for p53; thus, possible recurrence of SEIC was suspected. Tumor DNAs were microdissected from the uterine and peritoneal lesions and *p53* mutation analysis was done. SEIC and peritoneal carcinomas had distinct *p53* mutations that were mutually exclusive.

**Conclusions:**

The present case raised a concern about the difficulty of histologic staging for SEICs. Although SEICs confined to the uterine endometrium in most cases predict a good prognosis, microscopic metastasis to the peritoneum may not be detectable at hysterectomy. If secondary malignancies of a serous phenotype develop years later, comprehensive reexamination of SEIC is mandated, with the help of DNA analysis.

## Background

Uterine serous endometrial intraepithelial carcinoma (SEIC) is a unique malignancy that predominantly occurs in postmenopausal women [1, 2]. In most cases, patients with SEIC confined to the uterus have favorable prognoses [2, 3]; however, SEIC sometimes disseminates in the peritoneal cavity and/or metastasizes to distant sites [4, 5]. Primary peritoneal serous carcinoma is another malignancy that often involves gynecologic organs. The differential diagnosis includes metastasis from occult tubal intraepithelial serous carcinoma and SEIC. The majority of gynecologic malignancies of high-grade serous phenotype are histologically indistinguishable from peritoneal serous carcinoma. Multifocal occurrence should also be considered in peritoneal serous carcinoma [6]. Therefore, it is occasionally difficult to distinguish between peritoneal serous carcinomas primary lesion(s) and SEIC. Herein we describe an unusual case of peritoneal carcinoma in a patient previously diagnosed with SEIC.

## Case presentation

**Fig. 1:**
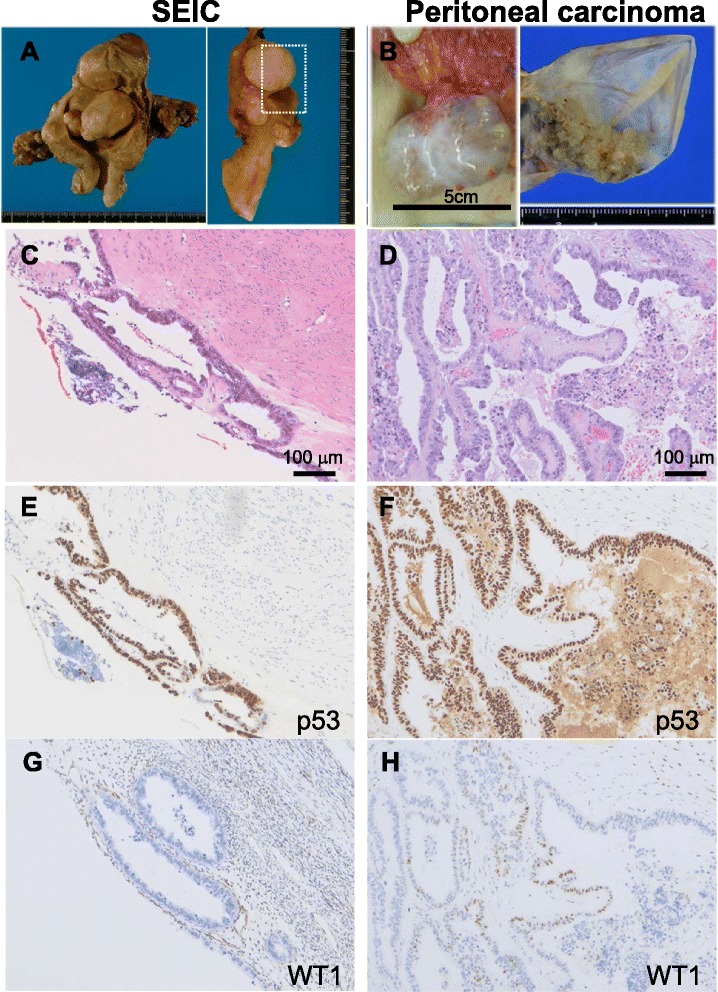
Macroscopic and microscopic features of serous endometrial intraepithelial carcinoma (SEIC) and peritoneal carcinoma. **a** The resected uterus had several leiomyomas occupying the uterine cavity (*left*). The cut surface of the vertical axis is shown (*right*). The surface in the dotted rectangle indicates the SEIC lesion. **b** The peritoneal carcinoma developed as a cyst on the surface of intestinal mesenchyme (*left*). Inside of the cyst, a papillary lesion was detected (*right*). **c** Hematoxylin-eosin (HE) staining of the SEIC in atrophic endometrium. Hyalinized submucosal leiomyoma is observed in the adjacent tissue. **d** HE staining of the peritoneal carcinoma. Proliferating papillary tumor cells with atypia are observed. **e**, **f** Immunostaining for p53 in the SEIC (E) and the peritoneal carcinoma (F). Both lesions show diffusely positive staining. **g**, **h** Immunostaining for WT-1 in the SEIC (G) and the peritoneal carcinoma (H). The SEIC is negative for WT-1 (G), whereas the peritoneal carcinoma shows focal positive staining (H). Serial staining sections are shown

A 71-year-old woman was referred to us due to elevated serum CA125 (184 U/mL). She had undergone hysterectomy and bilateral salpingo-oophorectomy 8 years previously due to SEIC and multiple leiomyomas (Fig. 1a). The SEIC lesion had been confined to the endometrium (FIGO Stage IA); however, peritoneal cytology was borderline (Class IIIb, atypical glandular cells). She had undergone periodic surveillance without postoperative chemotherapy. An elevated serum CA125 level was detected for the first time 8 years after hysterectomy and bilateral salpingo-oophorectomy. Computed tomography revealed a 5 × 4 cm cystic lesion in the abdominal cavity. A thorough medical workup denied any possible primary lesions in the body except for that in the peritoneum. The patient was suspected to either have primary peritoneal carcinoma or recurrent SEIC. Tumor debulking surgery was performed, and the cystic lesion and one disseminated nodule were resected. Inside of the cyst, papillary tumor proliferation was observed (Fig. 1b). The patient received 6 cycles of adjuvant chemotherapy, and complete remission has been achieved for 12 months.

**Fig. 2:**
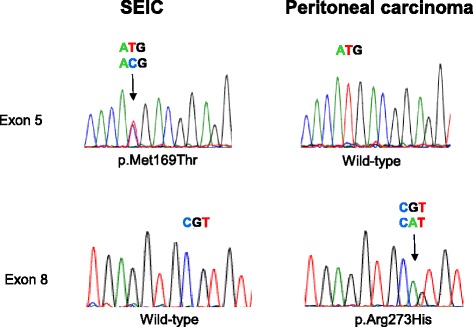
Distinct *p53* mutation patterns in SEIC and peritoneal carcinoma. (Upper sequences) *p53* exon 5 sequences of the SEIC (*left*) and the peritoneal carcinoma (*right*). A somatic mutation from ATG to ACG (*arrow*) was detected in the SEIC, predicting the amino acid change p.Met169Thr. The peritoneal carcinoma had a wild-type sequence at this position. (Lower sequences) *p53* exon 8 sequences of the SEIC (*left*) and the peritoneal carcinoma (*right*). A somatic mutation from CGT to CAT (*arrow*) was detected in the peritoneal carcinoma, predicting the amino acid change p.Arg273His. SEIC had a wild-type sequence at this position

Microscopically, peritoneal tumor cells had a morphologic appearance of high-grade serous carcinoma. The tumor cells proliferated in either gland-like or exophytic papillary patterns. Slit-like spaces were observed. Tumor cells had pleomorphic nuclei and eosinophilic cytoplasm, and the cyst wall was composed of a hyalinized membrane lined by one or a few epithelial cells. Although papillary tumors in the cyst arose continuously from the inner lining cells, it was difficult to determine by histology whether the peritoneal lesion was primary serous carcinoma or recurrent SEIC. Immunohistochemical analysis revealed that the peritoneal carcinoma was diffusely positive for cytokeratin 7, p53, and PAX 8, and was partially positive for WT-1, estrogen receptor (ER), and progesterone receptor (PgR).

We then reviewed the uterus and adnexa resected 8 years previously. Although the fallopian tubes and ovaries were not meticulously cut at 2-mm intervals, no malignant signs were present in the adnexa. The tubal mucosa and ovarian surface were normal in size and morphology, with atrophic features. Neither intraepithelial carcinoma/dysplasia nor disseminated carcinoma was detectable in the adnexa. The inner surface of the uterine endometrium was distorted due to submucosal leiomyoma. Neither gross endometrial lesions nor polyps were identified. Atrophic normal endometrium was detected adjacent to the SEIC, and the SEIC was distributed in a noninvasive manner. No lymphovascular space invasion was observed in the resected organs. Immunohistochemical analysis revealed that these glands were diffusely positive for p53 and PAX8, and negative for WT-1, ER, and PgR. The Ki-67 proliferation index was 70 %. The presence of a p53 signature with an elevated Ki-67 proliferation index was consistent with the histologic diagnosis of SEIC.

The patient did not have any medical history associated with familial cancer, such as hereditary breast ovarian carcinoma syndrome. Although 8 years had passed with a disease-free condition, possible metastatic carcinoma from the SEIC could not be completely denied. To investigate whether the two lesions were independent or represented metastasis from one site to another, SEIC and peritoneal carcinoma cells were microdissected, respectively. Written informed consent for molecular analysis of the surgical specimens was obtained from the patient. DNA of each lesion was extracted. Exons 5–8 of *p53* and exons 8–9 of *FBXW7* were amplified by polymerase chain reaction. The SEIC was revealed to have a *p53* mutation at p.M169T, whereas the peritoneal carcinoma had a mutation at p.R273H (Fig. 2). These mutations were mutually exclusive. No mutations in exons 8–9 of *FBXW7* were detected in either tumors.

## Discussion

Very little information is available regarding whether early-stage SEIC could potentially relapse 8 years after total hysterectomy and bilateral salpingo-oophorectomy [7]. Since both unifocal and multifocal serous carcinomas have been reported in gynecologic serous carcinomas, we considered a possible relapse in the present case. One report has been published in which a SEIC without stromal invasion resulted in distant metastasis 3 years after surgical intervention, in which both lesions shared an identical *p53* mutation [8]. Although differential immunostaining patterns of ER, PgR, and WT-1 between peritoneal serous carcinomas and SEIC are helpful for determining the origin, an argument exists against the utility of WT-1 for differential diagnosis [9, 10]. The WT-1 immunostaining pattern in the present peritoneal carcinoma was focal and weak; thus, more reliable information was required to make a conclusive diagnosis. Mutually-exclusive *p53* mutation patterns strongly suggested that the peritoneal lesion was a second primary cancer. If the SEIC had metastasized to the peritoneal cavity, both lesions would have shared identical mutation. Although secondary lesions potentially have different mutations, it is unlikely that the mutation of the primary lesion is normalized during metastasis. The present case alerted us to not associate the peritoneal serous carcinoma with the early-stage SEIC that occurred 8 years previously solely by histologic comparison.

It remains unclear whether the peritoneal carcinoma developed by chance or whether this patient was at risk of developing multifocal serous-type cancers. It is unlikely that she had mutations in the *BRCA* genes, because none of her siblings experienced cancers of the breast, ovary, or peritoneum. Since the peritoneal cytology had been Class IIIb at initial surgery, atypical peritoneal cells might have already been present 8 years previously. We also considered possible occult intraepithelial carcinoma in the adnexa. The lack of macroscopic signs of neoplasms in the ovary and fallopian tubes allowed us to perform usual sectioning of the adnexa. Lymph node staging was also not performed. Currently, no guidelines about whether complete sectioning of the adnexa should be performed or whether usual pathologic sectioning is sufficient for staging of SEICs exist. Although the possibility is very low that occult serous tubal/ovarian intraepithelial carcinoma disseminated and developed in the peritoneum 8 years after total resection, the present case alerted us to examine both adnexa and extra-pelvic lesions carefully in SEICs.

## Conclusion

We have described a case of metachronous peritoneal carcinoma that occurred 8 years after SEIC. Based on clinicopathologic findings and *p53* mutation patterns, we concluded that the two lesions were most likely independent. Limited information is available about possible recurrence of early-stage SEIC after 5 years of follow-up. Since microscopic metastases of SEICs in the adnexa and/or extra-pelvic organs lead to poor prognoses [3], gynecologists and pathologists should carefully investigate the possible presence of microscopic lesions in organs other than the uterus as well as lymphovascular space invasion in SEICs. Long-term follow-up will be beneficial not only for patients with advanced SEICs but also for those who are diagnosed with early-stage SEICs for a better understanding of the postoperative risks of serous carcinomas in the peritoneal cavity.

## Abbreviations

ER: Estrogen receptor
PgR: Progesterone receptor
SEIC: Serous endometrial intraepithelial carcinoma

## References

[1] Fadare O, Zheng W (2009). Insights into endometrial serous carcinogenesis and progression. Int J Clin Exp Pathol.

[2] Wheeler DT, Bell KA, Kurman RJ, Sherman ME (2000). Minimal uterine serous carcinoma: diagnosis and clinicopathologic correlation. Am J Surg Pathol.

[3] Hui P, Kelly M, O'Malley DM, Tavassoli F, Schwartz PE (2005). Minimal uterine serous carcinoma: a clinicopathological study of 40 cases. Mod Pathol.

[4] Gehrig PA, Groben PA, Fowler WC, Walton LA, Van Le L (2001). Noninvasive papillary serous carcinoma of the endometrium. Obstet Gynecol.

[5] Chan JK, Loizzi V, Youssef M, Osann K, Rutgers J, Vasilev SA (2003). Significance of comprehensive surgical staging in noninvasive papillary serous carcinoma of the endometrium. Gynecol Oncol.

[6] Muto MG, Welch WR, Mok SC, Bandera CA, Fishbaugh PM, Tsao SW (1995). Evidence for a multifocal origin of papillary serous carcinoma of the peritoneum. Cancer Res.

[7] Hou JY, McAndrew TC, Goldberg GL, Whitney K, Shahabi S (2014). A clinical and pathologic comparison between stage-matched endometrial intraepithelial carcinoma and uterine serous carcinoma: is there a difference?. Reprod Sci.

[8] Baergen RN, Warren CD, Isacson C, Ellenson LH (2001). Early uterine serous carcinoma: clonal origin of extrauterine disease. Int J Gynecol Pathol.

[9] Euscher ED, Malpica A, Deavers MT, Silva EG (2005). Differential expression of WT-1 in serous carcinomas in the peritoneum with or without associated serous carcinoma in endometrial polyps. Am J Surg Pathol.

[10] Hirschowitz L, Ganesan R, Mccluggage WG (2009). WT1, p53 and hormone receptor expression in uterine serous carcinoma. Histopathology.

